# Using Spectral Reflectance to Estimate the Leaf Chlorophyll Content of Maize Inoculated With Arbuscular Mycorrhizal Fungi Under Water Stress

**DOI:** 10.3389/fpls.2021.646173

**Published:** 2021-05-28

**Authors:** Jinhua Sun, Liu Yang, Xitian Yang, Jie Wei, Lantao Li, Erhui Guo, Yuhua Kong

**Affiliations:** ^1^College of Forestry, Henan Agricultural University, Zhengzhou, China; ^2^Henan Ecological and Environmental Monitoring Center, Zhengzhou, China; ^3^College of Resources and Environment, Henan Agricultural University, Zhengzhou, China

**Keywords:** arbuscular mycorrhizal fungi, leaf chlorophyll content, spectral reflectance, machine learning algorithms, water stress

## Abstract

Leaf chlorophyll content is an important indicator of the growth and photosynthesis of maize under water stress. The promotion of maize physiological growth by (AMF) has been studied. However, studies of the effects of AMF on the leaf chlorophyll content of maize under water stress as observed through spectral information are rare. In this study, a pot experiment was carried out to spectrally estimate the leaf chlorophyll content of maize subjected to different durations (20, 35, and 55 days); degrees of water stress (75%, 55% and 35% water supply) and two inoculation treatments (inoculation with *Funneliformis mosseae* and no inoculation). Three machine learning algorithms, including the back propagation (BP) method, least square support vector machine (LSSVM) and random forest (RF) method, were used to estimate the leaf chlorophyll content of maize. The results showed that AMF increased the leaf chlorophyll content, net photosynthetic rate (A), stomatal conductance (gs), transpiration rate (E), and water use efficiency (WUE) of maize but decreased the intercellular carbon dioxide concentration (Ci) of maize and atmospheric vapor pressure deficit (VPD) regardless of the water stress duration and degree. The first-order differential spectral data can better reflect the correlation between leaf chlorophyll content and spectrum of inoculated maize when compared with original spectral data. The BP model performed bestin modeling the maize leaf chlorophyll content, yielding the largest *R*^2^-values and smallest root mean square error (RMSE) values, regardless of stress duration. These results provide a reliable basis for the effective monitoring of the leaf chlorophyll content of maize under water stress.

## Introduction

Maize is one of the most important food crops in Asia. However, water stress is a main constraint of crop production at the global scale and is expected to increase in coming years (Lesk et al., 2016), and global crop yields are heavily affected by this constraint (Daryanto et al., 2016). Therefore, increasing the utilization efficiency of existing water resources is an effective way to mitigate agricultural water use limitations (Schewe et al., 2014).

Water stress affects the photosynthesis, transpiration, and water use efficiency (WUE) of plants and their absorption and utilization of water and nutrients and hinders the physiological and biochemical processes of plants (Kahil et al., 2015; Ortega-Farias et al., 2021). Plants can cope with water stress through their adaptive strategies (Pavithra and Yapa, 2018), and the symbiosis between arbuscular mycorrhizal fungi (AMF) and plants plays an important role in plants adaptations to water stress (Pavithra and Yapa, 2018). AMF canengage in mutualism with more than 80% of terrestrial plants in the world (Smith and Read, 2008). Plants provide carbohydrates to AMF, and AMF play a crucial role in the growth and nutrient uptake of host plants through beneficial physiological processes. Moreover, AMF improve plant performance under abiotic stresses such as drought (Pavithra and Yapa, 2018), pollution (Liu et al., 2018), or salinity (Arafat and He, 2011). AMF have been shown to improve plant absorption of water, increase photosynthetic pigments, stomatal conductance (Duc et al., 2018; Meddich et al., 2021), intercellular CO_2_ concentration, transpiration rate, root volume and diameter, and stimulate H^+^-ATPase activity and gene expression of plants in response to water stress (Cheng et al., 2021). *Bromus* species inoculated with AMF enhanced superoxide dismutase, peroxidase, catalase, and ascorbate peroxidase activities (Karimkhani et al., 2021) to help plants to tolerate water stress. In addition, inoculation with *F. mosseae* through the improvement of ionic and biochemical status of the plant can mitigate the detrimental effects of water-deficit stress on maize plants (Bahraminia et al., 2020). AMF increase water use and reduce oxidation damage by stimulating antioxidant activities (Pedranzani et al., 2016) and regulating water absorption and transport by aquaporin genes and endogenous hormones (Pedranzani et al., 2016; Quiroga et al., 2017; Cheng et al., 2021). Plant physiological phenomena are reflected in the healthy status of leaves. Leaf chlorophyll content is an important index for measuring plant photosynthesis and growth. However, the extraction and detection of leaf pigments by traditional chemical monitoring methods is tedious, destructive, discontinuous and time consuming. A spectro radio meter can be used to obtain plant spectral information through reflective rays at different wavelengths (700-1300 nm) with relatively high reflectivity observed in the near infrared region. The spectral reflectance of leaves in different wavebands represents different leaf characteristics of plants. Spectral reflectance in the visible and near infrared regions differs between plants under water stress and healthy plants. Spectro radio metric methods yield measurements more quickly, continuously and economically than traditional laboratory methods (Basayigit and Ozkul, 2015).

The vegetation indexes have been used to build inversion model of chlorophyll content of wheat, as the red edge parameters used to estimate chlorophyll content of plant under the stripe rust, water or salt stress have been investigated in many studies (Gu et al., 2008; Jiang et al., 2010; El-Hendawy et al., 2021). In the past, most models employed to estimate the leaf chlorophyll content have been based on linear regression method; however, this method cannot describe the complex relationship between modeling factors and dependent variables. Therefore, the modeling accuracy was not high. The use of machine learning algorithms is an effective way to express the complex relationship between the modeling factors and dependent variables (Saitta et al., 2011; Sonobe et al., 2021). The back propagation (BP) approach has been used to determine plant diseases (Saleem et al., 2019), and this methodology has potential in the analysis of hyperspectral reflectance data. The random forest (RF) method is a regression technique that combines numerous decision trees to classify or predict the value of a variable, and it has been used for estimating vegetation properties (Li et al., 2017; Chen et al., 2018) as well as for classification and regression (Biau and Scornet, 2015). Least square support vector machine (LSSVM) is a nonlinear system modeling method that has been proposed in recent years. LSSVM requires a small number of training samples and can approach nonlinear systems with high accuracy, and it has been widely used in many fields (Li et al., 2010; Mall and Suykens, 2015). However, studies involving the hyperspectral estimation of chlorophyll content in maize inoculated with AMF under drought stress are rare. In our research, pot experiments were conducted by controlling the amount of water, and the chlorophyll content of maize inoculated with AMF was monitored spectrally. Comparisons and analyses of the physiological characteristics and spectral response of AMF-inoculated and control maize under water stress were performed in this study. An inversion model of the chlorophyll content and multiple spectral variables was established. The objectives of this study were to (1) compare the chlorophyll content and other physiological characteristics of maize with or without inoculation under water stress, (2) determine the spectral response of AMF-inoculated and non-inoculated maize to water stress, and construct the best spectral estimation model of the chlorophyll content of maize under water stress.

## Methods

### Materials

Maize seeds were obtained from the Henan Academy of Agricultural Sciences; and *Funneliformis mosseae* was obtained from the Research Institute of Plant Nutrition and Resources in the Beijing Academy of Agriculture and Forestry Sciences and cultured in a microbial laboratory at Henan Agricultural University. River sand with poor nutrients was collected as a substrate and then air dried, passed through a 1 mm sieve and mixed thoroughly. The basic properties of the soil are listed in Table 1. The soil was sterilized at 121°C for 2 h, air dried and prepared for the pot experiment.

**TABLE 1:** Basic physical and chemical properties of sandy soil.

**pH**	**AP (mg/kg)**	**AN (mg/kg)**	**TN (%)**	**TC (%)**
7.78	1.66	28	0.107	0.891

*AP, available phosphorus content; AN, available nitrogen content; TN, total nitrogen content; TC, total carbon content.*

### Experimental Design

The experiment was conducted in a greenhouse at the Forestry College at Henan Agricultural University on August 15, 2019. The inoculation treatments included inoculation with *F. mosseae* and no inoculation; and the water stress treatments included (a) no stress, irrigation to 75% of the maximum water holding capacity of the soil (WS75%; achieved with 150 mL water per plant); (b) moderate stress, wherein irrigation was provided to 55% of the maximum water holding capacity of the soil (WS55%; achieved with 110 mL water per plant), and (c) severe stress, wherein irrigation was provided to 35% of the maximum water holding capacity of the soil (WS35%; achieved with 70 mL water per plant). The maize plants were subjected to the water stress treatments for 20, 35, or 55 days after sprouting (on August 20, 2019), in other words, the water stress starts on September 10, September 25, and October 15, 2019, respectively. With 20, 35, and 55 days after sprouting representing the seedling stage, three leaf stage and jointing stage, respectively, of maize growth. Each treatment was performed with 5 replicates. Therefore, a total of 90 pots of maize were established (2 (inoculation treatments) × 3 (water stress degree treatments) × 3 (water stress duration treatments) × 5(replicates)).

Maize was inoculated with *F. mosseae*at 50 g/pot (20 spores/g), and the non-inoculated maize was inoculated with the same amount of sterilized microbial inoculum. NH_4_NO_3_, KH_2_PO_4_, and K_2_SO_4_ were added to achieve 100 mg N/kg, 30 mg P/kg, and 150 K mg/kg, respectively, for fertilization. Before water stress, the plants were irrigated with 75% of the maximum water holding capacity of soil during the growth process, and the soil moisture meter (LB9007, QingDao, in China) was used for real-time monitoring. Maize seedling was irrigated in time every day to ensure that the soil moisture was maintained between70% and 75%. Maize seeds were disinfected with 10% H_2_O_2_ for 10 min, rinsed with water 2-3 times and then washed with deionized water4-5 times. Seeds were cultured in an incubator at 25-28°C for 24 h. Two seeds were sown in each pot (caliber × height × bottom diameter = 12 × 13 × 9.5 cm) before germination, and the best growing seedling was kept. The light intensity was controlled by 10 high-pressure sodium lamps, and the air temperature and humidity were controlled at 25°C and 20%, respectively, by an air-conditioning unit.

### Measurements

Leaf spectral collection and physiological measurement were carried out after each of the three water stress durations, and five pots were randomly selected from each water stress treatment.

### Spectrometric Determination

Leaf spectra were acquired with a Field Spec 3 ground spectrometer (ASD Company, United States) under dark conditions. The wave band range of the ground spectrometer was 350-1025 nm, and the spectral resolution was 3 nm. To accurately reflect the whole plant growth status, each pot was placed on a black workbench, and two leaves collected from each of the upper, middle and lower layers of the plant were measured. The halogen lamp used for the indoor test matched the spectrometer and was placed above the sample at an angle of 15° (the angle between the halogen lamp and ground normal) and 20 cm from the target. The field angle of the probe was 25°when observed from10 cm above the target (with the imaged object formed an angle with two edges of the maximum probe range). Each leaf was measured to obtain spectral information, and a whiteboard correction was conducted every 30 min before and during the measurements. Each leaf was lit from four directions during measurement, and five spectra were collected for each lighting direction to avoid errors of leaf curling. Reflectance data were obtained from the average spectral values from all directions.

### Physiological and Biochemical Determination

The chlorophyll contents of the leaves were determined using a SPAD-502 instrument (Konica Minolta, Japan). This device determines the relative chlorophyll content using dual wavelength optical absorbance measurements (at wavelengths of 620 and 940 nm) of leaf samples. The chlorophyll content was obtained from 5 selected points on each leaf during measurement, and the average value was taken to reflect the chlorophyll content of the whole leaf.

Photosynthetic parameters such as intercellular CO_2_ concentration (Ci), net photosynthetic rate (A), stomatal conductance (gs), transpiration rate (E), and water use efficiency (WUE) of maize leaves and water vapor pressure (VPD) were measured by a LI-6400 photosynthesis instrument (LI-COR, United States).

The drying method was adopted to measure the aboveground and belowground biomass of maize: the roots and shoots of maize were heated at 105°C for 30 min, dried at 75°C for 3 days, and weighed to determine the dry weight of maize. Mycorrhizal colonization was determined by the Phillips and Hayman methods (Phillips and Hayman, 1970).

### Determination of Soil Properties

The soil property analysis shown in Table 1 was performed using standard soil test procedures. The soil pH values were measured at a 1:2.5 soil/water ratio (w/v) via a pHmeter (Gao et al., 2015). The soil total carbon content (TC) and total nitrogen content (TN) were measured by Dumas combustion using an ElementarVario MAX CN analyzer with the combustion chamber set at 900°C and an oxygen flow rate of 125 mL min^–1^. The soil available phosphorus content (AP) was measured via the molybdenum antimony colorimetric method, and the soil available nitrogen content (AN) was determined by the alkali hydrolysis diffusion method (Bao, 1999).

### Data Analysis

#### Data Smoothing

During spectral data acquisition, the spectral curve may contain noise because the energy response varies according to the waveband and environment. Therefore, the spectral data must be smoothed. In this study, the Savitzky-Golay (SG) method was used to smooth the spectral curve. This method not only removes the high-frequency components but also retains the characteristic trends of the original curve, and the denoising effect of the SG method is better than that of other methods. The SG algorithm is a least-squares convolution smoothing method that is synthetically applied according to polynomial fitting order and smoothing degree; however, the error of the polynomial fitting curve is calculated by the first derivative. In this study, the SG smoothing method was realized by MATLAB 2014.

#### First-Order Differential Processing Method

Derivative spectroscopy can reduce the influences of light, atmospheric scattering, absorption and background on the spectral characteristics of targets and there by obviously enhance the correlations between the first-order differential processing of the original spectral reflectance and biochemical indexes. In this study, the smoothed original spectral curves were processed with the first-order differential method.

#### Modeling Methods

The LSSVM, BP, and RF models were used to estimate the chlorophyll content in the leaves through reflectance spectra, and they were implemented in MATLAB 2014.

The LSSVM method is widely used for complex nonlinear modeling. If the training sample set is (x_*i*_,y_*i*_), with i = 1, 2, …, n, then x∈R^*d*^ and y∈R. The main idea of the support vector machine (SVM) is as follows: first, a nonlinear mapping φ (⋅) is used to map the sample input space R^*d*^ to the feature space: φ (x) = (φ (x_1_), φ (x_2_),…, φ (x_*n*_)); next, the optimal decision function y = w^*T*^⋅φ (x)+b is constructed in this high-dimensional feature space, and then the model parameters w and b are determined based on the principle of structural risk minimization. The calculation formula of structural risk is as follows:

$$R=c\cdot R_{e\,m\,p}+1/2\,{||\text{w}||}^{2}$$

Where c is the normalization parameter and R_*emp*_ is the loss function, which is also called the empirical risk. The common loss functions include the first loss function, the quadratic loss function and the Hubber loss function. Different loss functions represent different SVM models; LSSVM is the SVM with the quadratic loss function. That is, R_*emp*_ = $\sum_{\text{i}}\xi_{\text{i}}^{2}$, where $\xi_{\text{i}}^{2}$ is the prediction error of the model to the training samples.

The training of sample data is performed via the BP method, and the weights and thresholds of the network are constantly modified, so that the error function decreases along the negative gradient direction and approaches the expected output. BP is a widely used neural network model that is primarily applied for function approximation, model recognition and classification, data compression and time series prediction. The BP network is composed of an input layer, a hidden layer and an output layer. The hidden layer can have one or more layers. The network adopts the S-type transfer function: f(x) = 1/(1 + e^x^). The error function E = ∑_i_(T_i_ + O_i_)^2^/2 (where T_*i*_ is the expected output and O_*i*_ is the calculated output of the network) can be minimized by adjusting the weights and thresholds of the network.

The RF method builds multiple decision trees and fuses them to obtain a more accurate and stable model, which is represented by the combination of the bagging idea and random selection features. The RF method constructs multiple decision trees. When a certain sample needs to be predicted, the prediction results of each tree in the forest for the sample are counted, and the final result is selected from these prediction results by the voting method. Randomness is reflected in two aspects: the selection features and selection of samples. Thus, each tree in the forest has both similarities and differences from other trees.

### Statistical Methods

Analysis of variance (ANOVA) was used to compare the effects of AMF among treatments on the mycorrhizal colonization, the growth and photosynthetic physiological progress of maize using the IBM SPSS 23.0 software program (SPSS Inc., Chicago, IL, United States). The least significant difference (LSD) test at the 0.05 probability level (p) was used to compare the means of the measured traits. Correlations between spectral and leaf chlorophyll content and two tailed test (significant level α was 0.05) were determined by the IBM SPSS 23.0 (SPSS Inc, Chicago, IL, United States). The figures were plotted using Origin 8.0 (OriginLab Corporation, Northampton, MA, United States).

## Results

### Mycorrhizal Colonization

**FIGURE 1:**
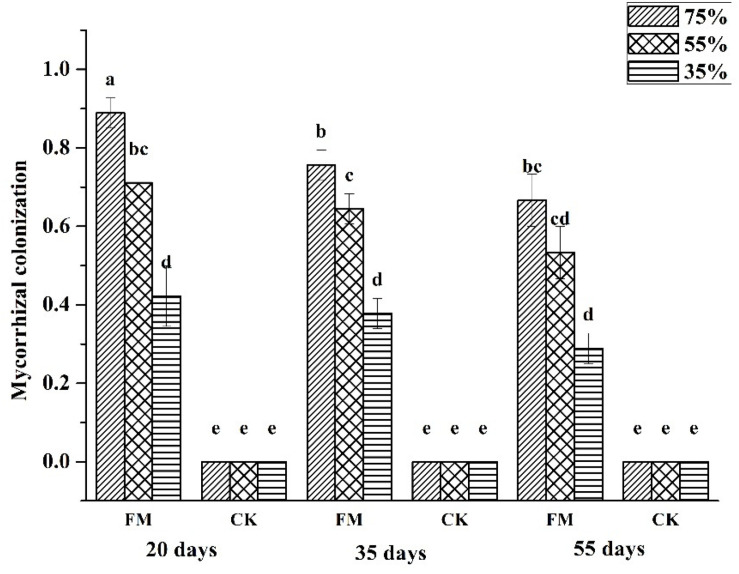
Mycorrhizal colonization of maize under different treatments. FM, inoculated with *F. mosseae*; CK, without inoculation.

Mycorrhizal colonization represents an intimate association between fungi and host plants. With increasing stress duration and degree, mycorrhizal colonization decreased gradually (Figure 1). Mycorrhizal colonization of inoculated maize was highest under the75% water supply regardless of stress duration and lowest under the35% water supply. Under the stress duration of 20 days, the mycorrhizal colonization of inoculated maize under a 75% and 55% water supply was increased significantly (*p* < 0.05) by 111.9% and 69% respectively, compared with that of inoculated maize under the 35% water supply. Under the stress duration of 35 days, the mycorrhizal colonization of inoculated maize under a 75% and 55% water supply was significantly increased (*p* < 0.05), by 100% and 29.2%, respectively, compared with that of maize under the 35% water supply; however, the mycorrhizal colonization of non-inoculated maize was zero, it may be because the maize grew on the sterile soil in an aseptic environment.

### Maize Growth

With increasing water stress, the chlorophyll content of the leaves in both the inoculated and non-inoculated maize decreased gradually; however, when the water stress duration was 20 days, the chlorophyll content of AMF-inoculated maize was4.33, 3.28 and 4.66 more than that of non-inoculated maize under a 75%, 55%, and 35% water supply, respectively. When the water stress duration was 35 or 55 days, the differences in chlorophyll content between AMF-inoculated and non-inoculated maize were not significant when a normal water supply was used; however, when the water stress duration was 35 days, the chlorophyll content of inoculated maize was significantly higher than that of non-inoculated maize when the water supply was 55% or 35%. Under the stress duration of 20 days, the aboveground biomass of AMF-inoculated maize was increased significantly, by 25.7%, compared with that of non-inoculated maize when the water supply was 55%. Moreover, the aboveground biomass of inoculated maize was greater than that of maize without inoculation under the other stress durations, although the differences were not significant (Table 2). The independent effects of stress duration, water stress degree, and inoculation were significant; however, the interaction effect of any two or three factors was not significant (Table 3).

**TABLE 2:** Physiological growth characteristics of maize under different treatments.

**Stress duration**	**Water stress**	**Inoculation**	**SPAD**	**Ci**	**A**	**gs**	**E**	**VPD**	**WUE**	**Aboveground biomass**
20 days	75%	FM	29.02 ± 0.49a	197.5 ± 2.2l	7.02 ± 0.21a	28.6 ± 0.95a	1.27 ± 0.08a	2.53 ± 0.07g	8.22 ± 1.41a	0.86 ± 0.14cd
		CK	24.69 ± 0.47d	258.5 ± 8.8j	6.85 ± 1.61ab	22.4 ± 1.1c	1.08 ± 0.33ab	2.78 ± 0.03f	6.32 ± 0.9ab	0.57 ± 0.18de
	55%	FM	27.76 ± 0.54b	210.2 ± 6.8k	6.35 ± 0.3b	25.6 ± 1.15b	1.13 ± 0.13ab	3.44 ± 0.45f	7.28 ± 0.85a	0.7 ± 0.08d
		CK	24.48 ± 2.97cd	276.7 ± 1.6i	6.42 ± 0.81ab	20.2 ± 1.53cd	0.88 ± 0.15 bc	4.38 ± 0.10e	5.8 ± 0.35b	0.52 ± 0.01e
	35%	FM	26.13 ± 0.49c	277.5 ± 2.5i	5.43 ± 1.43bc	21.2 ± 0.52c	0.72 ± 0.08cd	4.3 ± 0.22e	6.42 ± 1.28ab	0.52 ± 0.14de
		CK	21.47 ± 0.75d	304.7 ± 10.5h	3.9 ± 0.96cd	19.5 ± 0.75d	0.6 ± 0.05d	5.17 ± 0.19d	4.45 ± 0.6cd	0.35 ± 0.09e
35 days	75%	FM	24.92 ± 2.29cd	216.2 ± 4.2k	5.57 ± 0.35b	25.2 ± 0.74b	0.94 ± 0.03b	3.12 ± 0.06f	5.85 ± 0.48ab	1.57 ± 0.09b
		CK	21.52 ± 3.98de	265.2 ± 12.3ij	3.97 ± 0.20c	20.2 ± 1.53cd	0.72 ± 0.13d	4.12 ± 0.03ef	4.7 ± 0.2c	1.19 ± 0.25bc
	55%	FM	23.85 ± 1.05d	375.7 ± 21g	4.43 ± 0.15bc	21.3 ± 4.65c	0.81 ± 0.02b	4.43 ± 0.3e	4.28 ± 0.31cd	1.66 ± 0.3ab
		CK	20.34 ± 3.18e	387.5 ± 14.9g	3.15 ± 0.38d	17.9 ± 2.12d	0.72 ± 0.06cd	4.87 ± 0.04de	3.5 ± 0.41d	1.16 ± 0.78bc
	35%	FM	22.05 ± 0.92d	402 ± 19.5g	3.03 ± 0.73de	19.7 ± 1.61c	0.52 ± 0.03de	5.13 ± 0.03d	3.78 ± 0.51d	0.96 ± 0.05c
		CK	16.37 ± 1.7e	427.5 ± 14.1f	2.38 ± 0.32de	16.3 ± 1.26e	0.45 ± 0.09ef	6.35 ± 0.3c	2.57 ± 0.56e	0.93 ± 0.41cd
55 days	75%	FM	24.75 ± 0.44d	453.8 ± 1.5e	3.05 ± 0.15d	22.2 ± 0.58c	0.74 ± 0.05c	5.15 ± 0.05d	4.4 ± 0.41cd	2.37 ± 0.42a
		CK	21.76 ± 1.11d	483.2 ± 6.3b	2.52 ± 0.31de	18.3 ± 1.04de	0.61 ± 0.05d	6.15 ± 0.13c	3.37 ± 0.43d	1.58 ± 0.33ab
	55%	FM	22.65 ± 0.36d	459 ± 1.3d	2.25 ± 0.15e	19.3 ± 0.76d	0.6 ± 0.05d	6.27 ± 0.18c	3.62 ± 0.08d	1.71 ± 0.51ab
		CK	20.21 ± 3.58de	470.3 ± 1.8c	1.73 ± 0.25f	17.3 ± 1.53de	0.48 ± 0.06de	7.15 ± 0.05b	2.6 ± 0.20e	1.19 ± 0.33bc
	35%	FM	20.86 ± 2.34de	480.3 ± 7.4bc	1.48 ± 0.25fg	16 ± 1.32e	0.47 ± 0.03e	7.12 ± 0.03b	2.42 ± 0.46ef	1.5 ± 0.49bc
		CK	18.41 ± 1.25e	496.5 ± 2.5a	1 ± 0.26g	12.5 ± 2.00f	0.36 ± 0.05f	8.08 ± 0.08a	1.58 ± 0.24f	0.85 ± 0.7cd

*The value in the table are the mean value ± standard deviation of three repeated samples. Different lowercase letters represent significant differences at the level of 0.05. FM, inoculated with *F. mosseae*; CK, without inoculation; SPAD, leaf color value; Ci, intercellular carbon dioxide concentration; A, net photosynthetic rate; gs, stomatal conductance; E, transpiration rate; VPD, atmospheric vapor pressure deficit; WUE, water use efficiency.*

**TABLE 3:** *F*-test and *p*-Values for different treatments.

	**SPAD**	**Ci**	**A**	**gs**	**E**	**VPD**	**WUE**	**Aboveground biomass**
Stress time	0.00**	0.00**	0.00**	0.00**	0.00**	0.00**	0.00**	0.00**
F/n	26.99/2	223.46/2	169.50/2	45.73/2	65.00/2	128.9/2	132.77/2	31.78/2
Water stress degree	0.00**	0.00**	0.00**	0.00**	0.00**	0.00**	0.00**	0.00**
F/n	15.64/2	340.13/2	41.13/2	45.61/2	56.31/2	619.59/2	41.11/2	8.59/2
Inoculation	0.00**	0.00**	0.00**	0.00**	0.00**	0.00**	0.00**	0.00**
F/n	47.43/1	150.43/1	17.62/1	71.69/1	23.98/1	311.66/1	52.48/1	15.40/1
Stress duration × Water stress	0.93	0.00**	0.42	0.51	0.04*	0.26	0.74	0.28
F/n	0.22/4	128.70/4	1.00/4	0.84/4	2.90/4	1.38/4	0.50/4	1.32/4
Stress duration × Inoculation	0.41	0.00**	0.24	0.49	0.63	0.08	0.12	0.18
F/n	0.92/2	12.81/2	1.50/2	0.74/2	0.47/2	2.74/2	2.23/2	1.80/2
Water stress × Inoculation	0.66	0.00**	0.77	0.16	0.51	0.04	0.79	0.70
F/n	0.42/2	6.67/2	0.26/2	1.96/2	0.69/2	3.41/2	0.24/2	0.36/2
Stress duration × Water stress × Inoculation	0.91	0.02*	0.19	0.39	0.80	0.00**	0.97	0.89
F/n	0.24/4	3.48/4	1.60/4	1.06/4	0.41/4	5.86/4	0.13/4	0.29/4

SPAD, leaf color value; Ci, intercellular carbon dioxide concentration; A, net photosynthetic rate; gs, stomatal conductance; E, transpiration rate; VPD, atmospheric vapor pressure deficit;WUE, water use efficiency; F, *F*-test value; n, degrees of freedom. Superscript** indicates that the differences among treatments were significant at the level of 0.01; and superscript * indicates that the differences among treatments were significant at the level of 0.05.

### Photosynthetic Physiological Parameters of Maize

As shown in Table 2, water stress inhibited the photosynthesis and transpiration of maize, where as AMF inoculation alleviated the physiological damage of water stress to maize. Regardless of the water stress duration or watering conditions, Ci in the leaves of maize was significantly higher in non-inoculated maize than in AMF-inoculated maize (*p* < 0.05). As the degree of water stress increased, the Ci of the leaves of both inoculated and non-inoculated maize increased gradually. Under the stress duration of 20 days, the A of the inoculated maize was higher than that of the non-inoculated maize (*p* > 0.05). However, under a stress duration of 35 days, and a 75% and 55% water supply, the A of the inoculated maize was 1.6 and 1.28 times higher, respectively, than that of non-inoculated maize (*p* < 0.05). Under a stress duration of 55 days and a 55% water supply, the A of the inoculated maize was increased significantly, by 30%, compared with that of the non-inoculated maize (*p* < 0.05).

When the stress duration was 20 or 35 days, AMF significantly improved the gs of maize leaves regardless of the degree of water stress. The stomatal conductance of AMF-inoculated maize was significantly higher than that of non-inoculated maize regardless of water stress duration or degree (*p* < 0.05). With increasing stress duration, the transpiration effect of AMF-inoculated maize became greater than that of non-inoculated maize. When the stress duration was 55 days, under a 75% and 55% water supply, the E of AMF-inoculated maize was significantly higher than that of maize without inoculation, by 0.22 and 0.09 times, respectively (*p* < 0.05). Under the stress durations of 20 days, the atmospheric VPD was significantly lower (*p* < 0.05)in the inoculated maize than in the non-inoculated maize. Under the stress durations of 20 days, the WUE of leaves did not significantly differ between the inoculated and non-inoculated maize with a normal water supply, however, under a 75% and 55% water supply, the leaf WUE of the inoculated maize was significantly higher than that of the non-inoculated maize (*p* < 0.05).

Each type of treatment (stress duration, water stress degree, and inoculation) had a significant effect (*p* < 0.05)on the photosynthetic parameters of maize leaves. However, the interaction effect of any two or three treatment parameters was not significant for any parameter except for Ci, VPD, and E (Table 3).

### Spectral Characteristics of Maize Leaves

**FIGURE 2:**
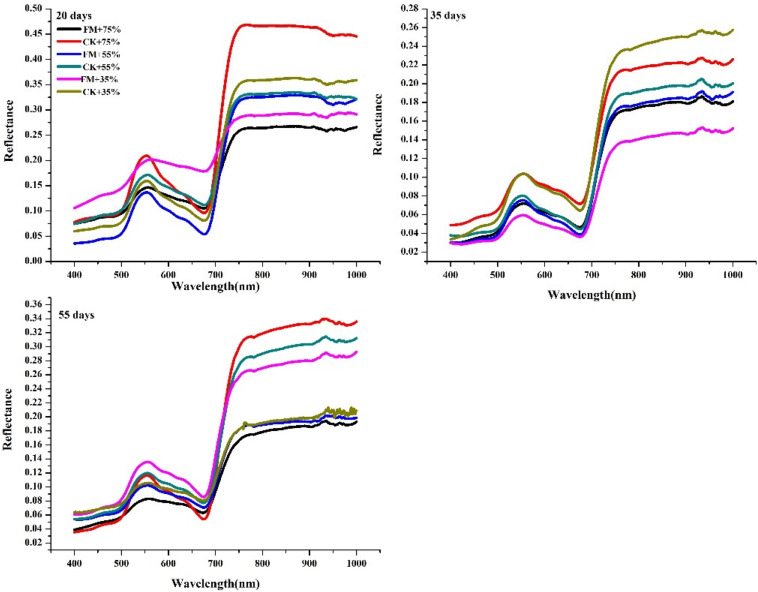
Spectra of maize leaves at each stress level and different inoculation durations. FM, inoculated with *F. mosseae*; CK, without inoculation.

At the three water stress durations, the general trend of the spectral curve of maize with different treatments was similar. When the stress duration was 20 or 35 days, at the green peak (550 nm), and red edge (700 nm), the leaf reflectance of non-inoculated maize was significantly higher (except under the 35% water supply) than that of inoculated maize (Figure 2). In non-inoculated maize under a 75% water supply, leaf reflectance was highest at wavelengths of 550 nm and 700 nm at a stress duration of 20 days whereas in non-inoculated maize with a 35% water supply, leaf reflectance was highest at wavelengths of 550 nm and 700 nm at a stress duration of 35 days. When the stress duration was 55 days, the leaf reflectance of inoculated maize with a 35% water supply was highest at a wavelength of 550 nm; and that of non-inoculated maize with a 75% water supply was highest at a wavelength of 700 nm. The highest reflectance of maize leaves under a stress duration of 20 days was higher than that of leaves under 35 and 55 days of stress at the wavelength of 550 and 700 nm (Figure 2).

### Correlation Analysis of Chlorophyll Content and First-Order Spectral Differential Variables in Maize

**FIGURE 3:**
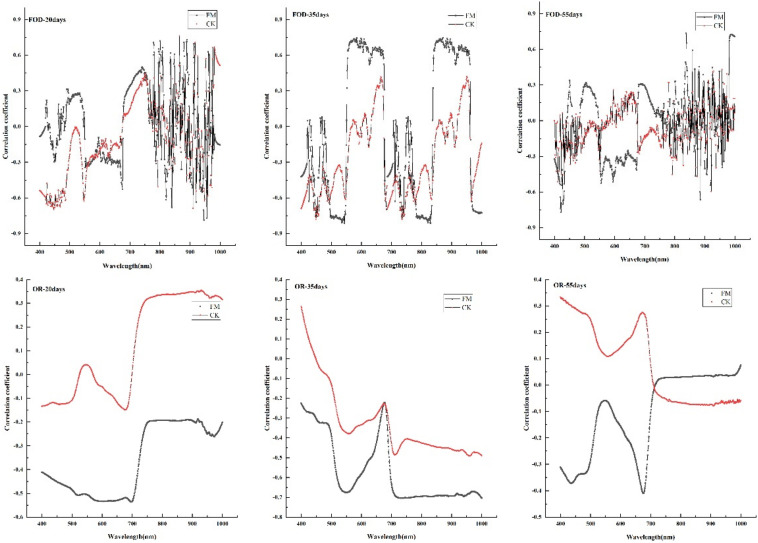
Correlation between spectral reflectance and leaf color value under different treatments. FM, inoculated with *F. mosseae*; CK, without inoculation. OR-20 days:water stress duration was 20 days with the original spectral data; OR-35 days: water stress duration was 35 days with the original spectral data; OR-55 days: water stress duration was 55 days with the original spectral data; FOD-20 days:water stress duration was 20 days with the first-order derivative of spectral data; FOD-35 days: water stress duration was 35 days with the first-order derivative of spectral data; FOD-55 days: water stress duration was 55 days with the first-order derivative of spectral data.

For the first-order derivative of spectral reflection, the spectral reflectance of inoculated maize leaves had larger correlation coefficients with the leaf chlorophyll content than that without inoculation under three water stress durations (Figure 3). When the stress duration was 20 or 55 days, the correlation coefficients between the first-order differential reflectance and the chlorophyll content of inoculated maize showed unstable performance at the range of 800-1000 nm especially, but during 400-800 nm, the maximum correlation coefficients values are in the inoculated maize at 550 nm and 700 nm, respectively (α < 0.05). When the stress duration was 35 days, the correlation coefficients between the first-order differential reflectance and the chlorophyll content of inoculated maize showed larger than that without inoculation at two peak areas (the wavelengths of 550-700 nm and 850-950 nm; Figure 3) (α < 0.05).

For the original spectral data, there was a negative correlation between leaf chlorophyll content and spectral reflectance of inoculated maize under three water stress durations, but a positive correlation between that of non-inoculated maize before 500 nm under the stress duration of 35 days and before 700 nm under the stress duration of 55 days. There are two peaks of correlation coefficient between leaf color value and spectral reflectance of inoculated maize at 550 and 700 nm were larger than that of non-inoculated maize under the stress duration of 35 or 55 days (Figure 3) (α < 0.05).

Compared with the original spectral data, the first-order differential processing can better reflect the correlation between leaf color value and spectrum of inoculated maize.

### Hyperspectral Estimation Model of the Chlorophyll Content of Maize and Its Validation

**FIGURE 4:**
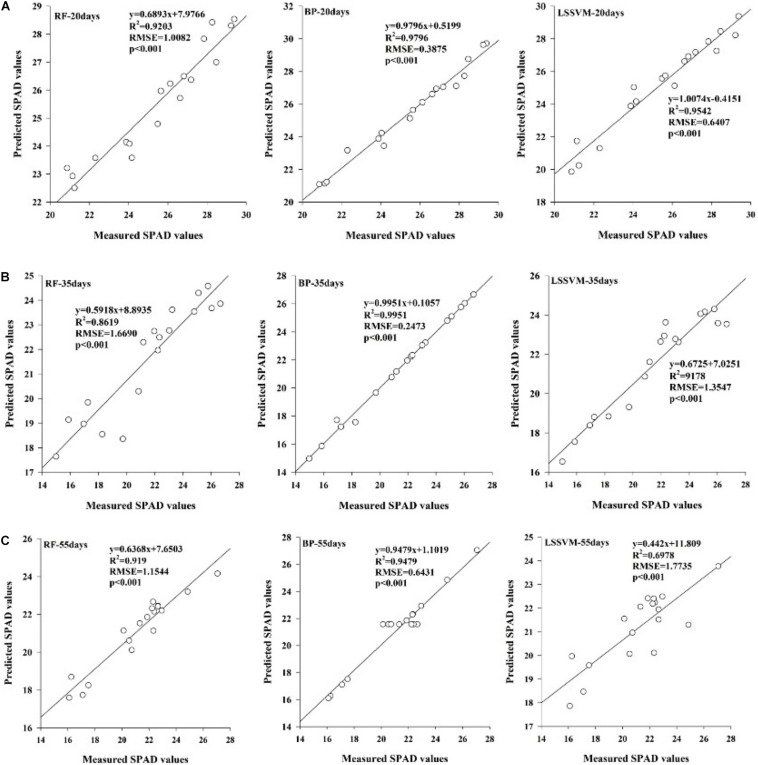
Relationships of the measured SPAD values and predicted values based on the three machine learning algorithms under water stress durations of 20 days **(A)**, 35 days **(B)**, and 55 days **(C)**.

To better reflect the chlorophyll content of inoculated maize under drought stress, the results of correlation analysis of the first-order differential spectral data were used to establish, an estimation model of the first-order differential values from sensitive bands corresponding to chlorophyll content. The LSSVM, RF, and BP nonlinear models based on machine learning were used. A regression analysis was carried out between the predicted and measured values, and the estimation accuracy of the models was evaluated by the coefficient of determination (*R*^2^) values, root mean square error (RMSE)values and *p*-Value of the LSSVM, RF and BP models established with different spectral variables that were significant at the 0.001 level (Figures 4A–C). The results indicated that all of these models could be used to estimate the chlorophyll content of inoculated maize under drought stress. The BP model was better than the others achieving the largest *R*^2^-values (*R*^2^ = 0.9796 for the 20 days duration, *R*^2^ = 0.9951 for the 35 days duration, and *R*^2^ = 0.9479 for the 55 days duration) and smallest RMSEs (RMSE = 0.3875 for the20-day duration, RMSE = 0.2473 for the35-day duration, and RMSE = 0.6431 for the 55-day duration) regardless of the stress duration.

## Discussion

Water stress is a worldwide problem that affects the growth, yield and physiological processes of crops (Begum et al., 2019). AMF symbiosis has been reported to enhance plant tolerance to water stress (Duc et al., 2018; Zhang et al., 2018). Inoculation with *F. mosseae*has been shown to enhance plant dry weights compared to those of non-mycorrhizal plants under control and water-deficit stress conditions (Bahraminia et al., 2020). In this study, AMF colonization was negatively affected by water stress, possibly because of declines in spore germination and growth, reductions in the number of AMF structures, the inhibition of hyphal growth and expansion in soil (Salloum et al., 2018), or reductions in the supply of carbohydrate by host plants (Tyagi et al., 2017). However, AMF inoculation improved the growth of maize regardless of the stress degree and duration, possibly because the AMF facilitated maize absorption of available phosphorus and nitrogen under water stress (Ghorchiani et al., 2018). Moreover, with increasing degree of water stress, the stress-mitigation function of the AMF became more obvious (Pavithra and Yapa, 2018). The AMF may have reduced the damage of water stress to plant growth by changing plant physiological processes (Pavithra and Yapa, 2018) and radial root water transport (Quiroga et al., 2019).

Photosynthesis is an important indicator of physiological sensitivity to water stress (Chaves et al., 2009). In our study, water stress inhibited photosynthesis in maize. Similar results have been reported by others (Hazrati et al., 2016; Ahanger et al., 2017). Many studies have shown that water stress reduces A, gs and E (Yan et al., 2016; Hura et al., 2007). The reduced photosynthesis under water stress is due to reductions in the coupling factor and production of ATP (Tezara et al., 1999). AMF improved the photosynthesis of maize under the different treatments in this study as well as previous studies (Sánchez-Blanco et al., 2004; Khalvati et al., 2005). In this study, in maize under water stress, Ci and VPD decreased and the A, gs, E and WUE increased in AMF-inoculated maize relative to non-inoculated maize. Increased stomatal functioning enhances CO_2_ entry into the leaf tissues and its successful assimilation was observed in AMF-inoculated plants, such results have also been reported in AMF-inoculated wheat (Zhou et al., 2015). The high photosynthetic rates of A MF-inoculated plants under water stress could be explained by the increased production of photosynthetic pigments and a higher carboxylation efficiency relative to that of non-inoculated plants (Sánchez-Blanco et al., 2004; Zhu et al., 2014).

Though the leaf chlorophyll content of maize was measured by SPAD instrument in a real time, fast and non-destructive way in this study, it was a relative value with the limitation that not taking into consideration of the biomass (Lei et al., 2019). In our study, AMF inoculation increased the leaf chlorophyll content of maize under water stress, consistent with there port of other researchers (Begum et al., 2019). It may be due to the key role of Mg in chlorophyll synthesis, and Mg uptake increased in mycorrhizal plants (Mathur et al., 2018). The spectral reflectance of leaves is closely related to the leaf surface characteristics, leaf thickness, water content, and the contents of chlorophyll and other pigments (Feng et al., 2004). The wavelength region (500 to 900 nm) contains wavelengths with pigment absorption features (Merzlyak et al., 2003) as well as the red-edge (700 to 750 nm) (Mutanga and Skidmore, 2007). In our study, at the green peak (550 nm) and red edge (700 nm), The spectral reflectance of maize without inoculation under water stress was much higher than that of AMF-inoculated maize, it may be due to the more water utilization rate and chlorophyll content of inoculated maize compared to that without inoculation (Begum et al., 2019). The spectra lreflectance of maize leaves decreased with water stress duration, potentially due to greater damage of water stress to the leaves of maize at the seedling stage than at the later growth stages. The results of this study indicated that hyperspectral technology can be used to monitor changes in chlorophyll content in maize inoculated with mycorrhizae and exposed to water stress.

In the present study, compared with the original spectra, the first-order differential reflectance spectra better reflected the chlorophyll content of leaves at 400-800 nm. In other study, the correlation curve between the original spectrum and the chlorophyll relative content value was the best between the wavelengths 509-650 nm. The correlation between the first derivative spectrum and chlorophyll relative content value was the best and most stable at 450∼500 nm (Zhu et al., 2020). It can be explained by that the original spectrum can eliminate the influence of background noise on the spectrum, and reduce the scattering and absorption of light by atmosphere in the process of spectrum acquisition, after first-order differential processing (Mahlein et al., 2012; Manzo et al., 2013). The correlation coefficient between leaf color value and first-order differential spectral reflectance of inoculated maize at 550 and 700 nm were larger than that of non-inoculated maize under the stress duration of 35 or 55 days. The results varied in different conditions and environments. Hunt et al. (2013) found that the correlation between chlorophyll content and spectral reflectance at the canopy scale was strong in the visible region (400-760 nm). Some study showed that the red edge slope is the maximum reflectance in the red band, which can better reflect the chlorophyll content of plants and is often closely related to the photosynthetic rate of plants (Verrelst et al., 2010).

Our results demonstrated that all the models, constructed using the LSSVM, RF, and BP methods, could be used to estimate the chlorophyll content of inoculated maize under drought stress. The BP model showed the highest *R*^2^ and slope and the lowest RMSE regardless of the water stress duration. BP model was more stable and accurate than others at estimation of leaf chlorophyll content (Shao et al., 2018). The best estimating model for the relationship between the leaf relative chlorophyll content and the reflectance spectra was the partial least squares (PLS) in the field experiment about winter wheat (Zhang et al., 2016). It may due to BP model was suitable for estimating the chlorophyll content in greenhouse experiment.

## Conclusion

Arbuscular mycorrhizal fungi treatment increased the leaf chlorophyll content, A, gs, E, and WUE of maize but decreased the Ciofmaize and VPD regardless of the water stress duration or degree. The first-order differential reflectance of inoculated maize leaves was more significantly correlated with the chlorophyll content of maize than was the original spectral reflectance. Of the three machine learning models, the BP model achieved the largest coefficient of determination and smallest RMSE regardless of the stress duration. Thus, the BP model represented the optimal method of estimating the leaf chlorophyll content of inoculated maize.

## Data Availability Statement

The original contributions presented in the study are included in the article/supplementary material, further inquiries can be directed to the corresponding author/s.

## Author Contributions

LY supervised the work, contributed to data interpretation, and writing and revision of the manuscript. JS designed and performed the investigation, analyzed the data, contributed to data interpretation, and drafted the manuscript. XY provided the test site and revised the manuscript. JW and LL provided experimental instruments and modification of manuscript. EG and YK contributed to modification of manuscript. All authors contributed to the article and approved the submitted version.

## Conflict of Interest

The authors declare that the research was conducted in the absence of any commercial or financial relationships that could be construed as a potential conflict of interest.
